# CCNE1 amplification is associated with poor prognosis in patients with triple negative breast cancer

**DOI:** 10.1186/s12885-019-5290-4

**Published:** 2019-01-21

**Authors:** Zi-Ming Zhao, Susan E. Yost, Katherine E. Hutchinson, Sierra Min Li, Yate-Ching Yuan, Javad Noorbakhsh, Zheng Liu, Charles Warden, Radia M. Johnson, Xiwei Wu, Jeffrey H. Chuang, Yuan Yuan

**Affiliations:** 1 The Jackson Laboratory for Genomic Medicine, Farmington, CT USA; 2 City of Hope Comprehensive Cancer Center and Beckman Research Institute, 1500 E. Duarte Road, Duarte, CA 91010 USA; 3 Genentech, Inc., Oncology Biomarker Development, South San Francisco, CA USA

**Keywords:** Triple negative breast cancer, CCNE1, Amplification

## Abstract

**Background:**

Triple negative breast cancer (TNBC) is aggressive with limited treatment options upon recurrence. Molecular discordance between primary and metastatic TNBC has been observed, but the degree of biological heterogeneity has not been fully explored. Furthermore, genomic evolution through treatment is poorly understood. In this study, we aim to characterize the genomic changes between paired primary and metastatic TNBCs through transcriptomic and genomic profiling, and to identify genomic alterations which may contribute to chemotherapy resistance.

**Methods:**

Genomic alterations and mRNA expression of 10 paired primary and metastatic TNBCs were determined through targeted sequencing, microarray analysis, and RNA sequencing. Commonly mutated genes, as well as differentially expressed and co-expressed genes were identified. We further explored the clinical relevance of differentially expressed genes between primary and metastatic tumors to patient survival using large public datasets.

**Results:**

Through gene expression profiling, we observed a shift in TNBC subtype classifications between primary and metastatic TNBCs. A panel of eight cancer driver genes (*CCNE1, TPX2, ELF3, FANCL, JAK2, GSK3B, CEP76,* and *SYK*) were differentially expressed in recurrent TNBCs, and were also overexpressed in TCGA and METABRIC. *CCNE1* and *TPX2* were co-overexpressed in TNBCs. DNA mutation profiling showed that multiple mutations occurred in genes comprising a number of potentially targetable pathways including PI3K/AKT/mTOR, RAS/MAPK, cell cycle, and growth factor receptor signaling, reaffirming the wide heterogeneity of mechanisms driving TNBC. *CCNE1* amplification was associated with poor overall survival in patients with metastatic TNBC.

**Conclusions:**

*CCNE1* amplification may confer resistance to chemotherapy and is associated with poor overall survival in TNBC.

**Electronic supplementary material:**

The online version of this article (10.1186/s12885-019-5290-4) contains supplementary material, which is available to authorized users.

## Background

Triple negative breast cancer (TNBC) is an aggressive type of breast cancer (BC) characterized by a high rate of recurrence and poor overall survival upon cancer metastasis [1, 2]. Hormone-receptor positive (HR+) BCs and HER2-positive (HER2+) BCs harbor well-defined and targetable biomarkers. Despite the identification of at least four molecular TNBC subtypes, including basal-like 1 (BL1), basal-like 2 (BL2), mesenchymal (M), and luminal androgen receptor (LAR) [3–5], these subtypes have not proven clinically useful due to the intrinsically complex and heterogeneous biology of TNBCs. With the exception of PARP inhibitors for patients with *BRCA1/2* germline mutations, and unlike HR+ or HER2+ BCs, there is no effective targeted therapy available to treat TNBCs.

In addition to tumor heterogeneity, the biology of TNBC is further complicated by tumor evolution through selective pressure. The molecular evolution of TNBC as the result of chemotherapy and/or radiation-induced selection pressure is well recognized but poorly understood [6]. Molecular discordance between primary and metastatic TNBCs has been observed, but the degree of biological heterogeneity has not been fully explored [7].

Recurrent/resistant BC may differ from primary tumors at multiple levels. Several previous studies demonstrated phenotypic discordances between primary and metastatic tumors in the standard-of-care biomarkers estrogen receptor (ER), progesterone receptor (PR), and HER2 [8, 9]. Discordance of other molecular markers such as *PIK3CA* mutations was also identified [9, 10]. Cyclin E1 (*CCNE1*), along with its catalytic subunit CDK2, plays a critical role in cell cycle regulation, DNA replication, chromosome segregation, and the G1 to S-phase transition [11, 12]. *CCNE1* amplification is associated with primary treatment resistance in high-grade serous ovarian carcinomas (HGSCs) and co-amplification of *TPX2* with *CCNE1* was common [13, 14]. *CCNE1* amplification is also associated with resistance to HER2-targeted therapy in HER2+ BC [15]. However, the role of *CCNE1* in TNBC is not well understood.

Genomic and transcriptomic discordance between primary and metastatic tumors have the potential to reveal novel drivers of metastatic progression, and aid in the selection of late-line therapies [16]. Genomic sequencing has revealed subclonal diversity of primary BCs and chemotherapy-resistant BCs in experimental models [17], but such findings have not been shown in patient tumor samples. Available large-scale public genomic databases from The Cancer Genome Atlas (TCGA) project [18] and the Molecular Taxonomy of Breast Cancer International Consortium (METABRIC) [19] have significantly improved our understanding of breast cancer tumor biology. These data sets contain only newly diagnosed and chemotherapy naïve tumors as opposed to complementary longitudinal primary and metastatic samples. Therefore, genomic characterization of longitudinal samples has the potential to identify novel driver mutations and therapeutic targets.

The primary goal of this study is to characterize the genomic changes between paired primary and metastatic TNBCs through sequencing and mRNA expression profiling, and to identify genomic alterations which may contribute to chemotherapy resistance.

## Methods

### Cohort of paired primary and recurrent/metastatic TNBC specimens

Paired primary and recurrent TNBC specimens were identified through an IRB-approved protocol from patients with recurrence between 2002 and 2015. Eligible patients had the following features: stage I - III TNBC; at least one tumor biospecimen available from initial surgery or biopsy; at least one specimen available from a recurrent/metastatic disease; and treatment and clinical outcome data available for chart review. Two cohorts of patients were studied: “Pilot-TNBC” (*n* = 10) and “Discovery-TNBC” (*n* = 55). All pathology samples were formalin-fixed and paraffin-embedded (FFPE). Demographic data such as age, gender, date of birth, date of diagnosis, date of relapse, and date of death or last follow-up (if applicable) were obtained for *CCNE1* amplified patients (*n* = 13, 6 from “Discovery-TNBC”, and 7 from clinical report). Disease characteristics such as tumor grade, TNM stage, and ER/PR/HER2 status, as well as treatment variables including surgery, chemotherapy, and radiation therapy were also obtained.

### Gene expression profiling of paired TNBCs

Paired primary and recurrent TNBC samples were profiled using the Affymetrix Human GeneChip® 2.0 in the “Pilot-TNBC” cohort. Robust multi-array averages (RMA [20]) were calculated from mRNA expression, summarized at the gene symbol level (23,945 gene symbols) based upon probe-set annotations (instead of transcript clusters) with the Affymetrix Power Tools software [21]. Messenger RNA (mRNA) expression across control probe transcript clusters was also summarized (27,293 control probes). Gene symbol annotations for probe sets were downloaded from the Affymetrix website. When comparing expression between primary and recurrent samples, *p*-values were calculated using the limma R package [22] and false discovery rate (FDR) values were calculated using the method of Benjamini and Hochberg [23]. The number of differentially expressed genes was maximized by considering genes with RMA > 2 in at least 50% of samples, |fold-change| > 1.5 between averaged recurrent and primary tumors, and FDR < 0.25. The ratio of gene expression between paired recurrent and primary TNBCs was reported as fold-change. Heat maps and clustering of differentially expressed genes were created using the ‘seaborn’ package under Jupyter Notebook Python 3. For better visualization, we only reported fold changes between − 10 and 10. TNBC subtyping was performed using the online TNBC Type tool from Vanderbilt University Medical Center [24]. Of the “Discovery-TNBC” cohort (*n* = 55), RNA isolation and expression profiling were performed for 35 paired primary-metastatic specimens. Total RNA was isolated from FFPE specimens using the QIAGEN miRNeasy FFPE kit. RNA integrity (RIN) and DV200 scores were determined using an Agilent Bioanalyzer. Per Illumina protocol, TruSeq RNA Access Library Preparation was performed, and libraries were enriched for mRNA fraction by positive selection, prior to paired-end 2 × 100 sequencing on the Illumina HiSeq. Using the R package “edgeR version 3.20.9” [25, 26], raw transcript counts were converted to log2-counts per million (log2CPM), dataset-wide lowly- or non-expressed genes were removed, and transcript expression was normalized to ensure similar expression distributions across the dataset.

### Targeted exome sequencing of TNBCs

Genomic alterations in FFPE specimens from primary and recurrent TNBCs were detected using the FoundationOne™ sequencing panel. FoundationOne™ identifies base substitutions, insertions and deletions (indels), amplifications with copy number ≥ 6, and rearrangements. The FoundationOne™ sequencing panel version used herein included the entire coding regions of 395 cancer-related genes, and select introns of 31 genes that are rearranged or altered in cancer, capable of achieving a median sequencing depth greater than 500X [27, 28].

### Comparison of gene expression using public databases

Gene expression of 1904 samples from METABRIC (299 TNBC and 1605 non-TNBC) using Illumina HT-12 arrays were downloaded [19]. Whole-transcriptome sequences of 1091 primary breast cancer (115 TNBC and 976 non-TNBC) and 112 normal cases in TCGA were downloaded in the format of ‘illuminahiseq_rnaseqv2-RSEM_genes_normalized (MD5)’ from FireBrowse [18]. Boxplots for gene expression comparisons in METABRIC [19] and TCGA [18] datasets were created using the R “ggplot” package. TCGA data were transformed to log2 of the gene expression value plus 1, to make them the same scale as the METABRIC gene expression data. *P*-values were independently calculated using Wilcoxon and ANOVA tests to compare between two, and among multiple groups, respectively.

### Statistical analysis

Kaplan-Meier (K-M) curves were generated for overall survival (OS) and relapse free survival (RFS), where OS is defined as time from surgery to death, and RFS is defined as time from surgery to disease recurrence. The log-rank test was used to examine survival (OS or RFS) difference based on *CCNE1* copy number alteration (CN ≥ 6 vs. CN < 6), or CCNE1 mRNA expression (median gene expression as a bifurcation).

## Results

### Patient characteristics and treatment history

The clinical characteristics, pathological features, treatment histories, and survival of the “Pilot-TNBC” cohort are described in Table 1. The majority of the tumors were infiltrating ductal carcinomas (IDC) (80%, 8/10), stage I-II (70%, 7/10), and the patients received standard-of-care chemotherapy with anthracycline and/or a taxane-containing regimen. RFS ranged from 2 to 39 months, and overall survival ranged from 9 to 113 months. Molecular subtypes according to the Lehmann/Pietenpol classification were determined: BL-1 (*n* = 4), BL-2 (*n* = 1), M (*n* = 3), and LAR (*n* = 2). Four of ten TNBC pairs displayed a shift in the molecular subtype between the primary and metastatic specimens.

**Table 1:** Patient characteristics in the “Pilot-TNBC” cohort

Pt ID	Spec# 1	Spec# 2	Histology	Age range	Stage	Lehmann subtypes	(Neo)adjuvant chemo	Radiation	RFS (months)	OS (months)
1	Breast	Lung Met	IDC	60–69	II	BL2 → BL2	AC-T	Yes	34	113
2	Breast	Bone Met	IDC	50–59	II	LAR→LAR	AC	No	17	60
3	Breast	LN Met	IDC	40–49	II	BL1 → BL1	AC	No	39	92
4	LN	Liver Met	IDC	40–49	III	BL1 → LAR	Carbo/Taxol	Yes	10	20
5	Breast	Skin Met	IDC	30–39	III	M → M	AC	Unknown	2	9
6	Brain	Soft tissue Met	IDC	50–59	II	M → M	TAC	No	31	67
7	Breast	LN Met	IDC	50–59	I	BL1 → BL2	Declined	No	26	46
8	Endometrium	LN Met	IDC	30–39	III	M → LAR	TC	Unknown	11	58
9	Breast	Contralateral breast Met	ILC	40–49	II	LAR→BL2	AC-T	Yes	8	23
10	Breast	Brain Met	Metaplastic	50–59	II	BL1 → BL1	AC-T	Unknown	16	58

*IDC* invasive ductal carcinoma, *LN* lymph node, *RFS* relapse-free survival (surgery to first relapse), *OS* overall survival (surgery to death), *ILC* invasive lobular carcinoma, *AC* adriamycin/cyclophosphamide, *AC-T* adriamycin/cyclophosphamide - > paclitaxel, *Carbo/taxol* carboplatin/paclitaxel, *TAC* docetaxel/adriamycin/cyclophosphamide, *TC* docetaxel /cyclophosphamide, *Met* metastatic tumor, *BL1* basal-like 1, *BL2* basal-like 2, *M* mesenchymal, *LAR* luminal androgen receptor, *Spec* specimen

### Genomic and transcriptomic profiling of paired TNBCs in the “Pilot-TNBC” cohort

**Fig. 1:**
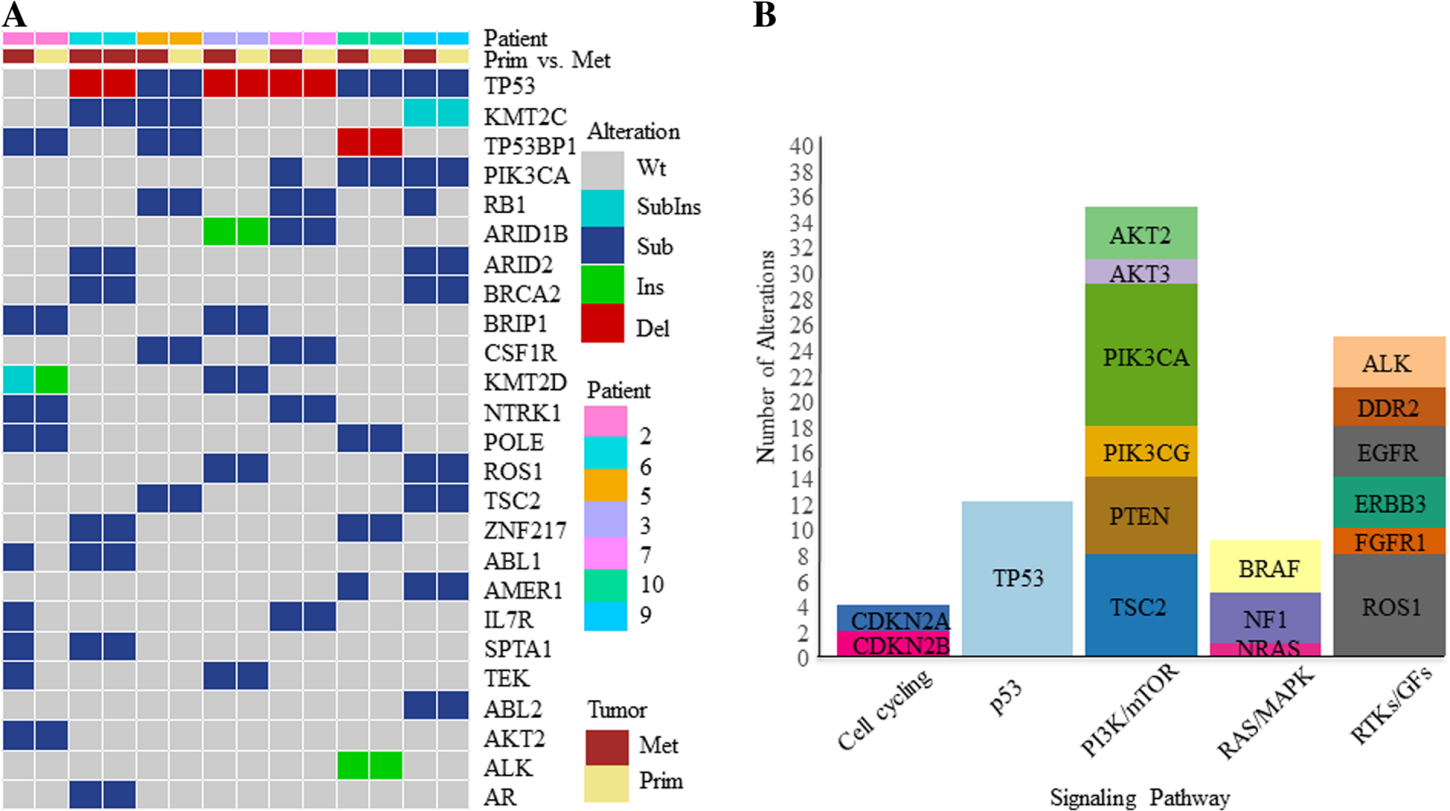
Genomic profiling of the “Pilot-TNBC” cohort. **a** Tile plot illustrating patients (columns) ordered by primary/metastatic status and by mutation frequency for the top 25 mutated genes; **b** Stacked bar plot illustrates the frequency of mutations observed in specific cellular signaling pathways: cell cycling, p53, PI3K/mTOR, RAS/MAPK, and RTK/GF signaling pathways [29]. Variants of unknown significance (VUS) were included for these analyses. Wt, wild type; SubIns, substitution and insertion; Sub, substitution; Ins, insertion; Del, deletion, Met, metastatic tumor; Prim, primary tumor

In “Pilot-TNBC,” FoundationOne™ sequencing was successful in 7 paired specimens (*n* = 14) (Fig. 1a). A total of 339 genomic alterations including 73 known mutation/amplification and 266 variants of unknown significance (VUS) were identified. Genomic alterations were identified in the following signaling pathways: cell cycle, p53, PI3K/mTOR, RAS/MAPK, and RTK/GF (Fig. 1b) [29]. There were no *CCNE1* mutations or amplifications detected in the “Pilot-TNBC” cohort. Detailed genomic alteration data is listed in Additional file 1: Table S3. In addition, there was significant inter-patient genomic heterogeneity but little intra-patient variability. These findings not only confirm the genomic heterogeneity of TNBCs, but also highlight the genomic stability of these tumors over the course of time.

**Fig. 2:**
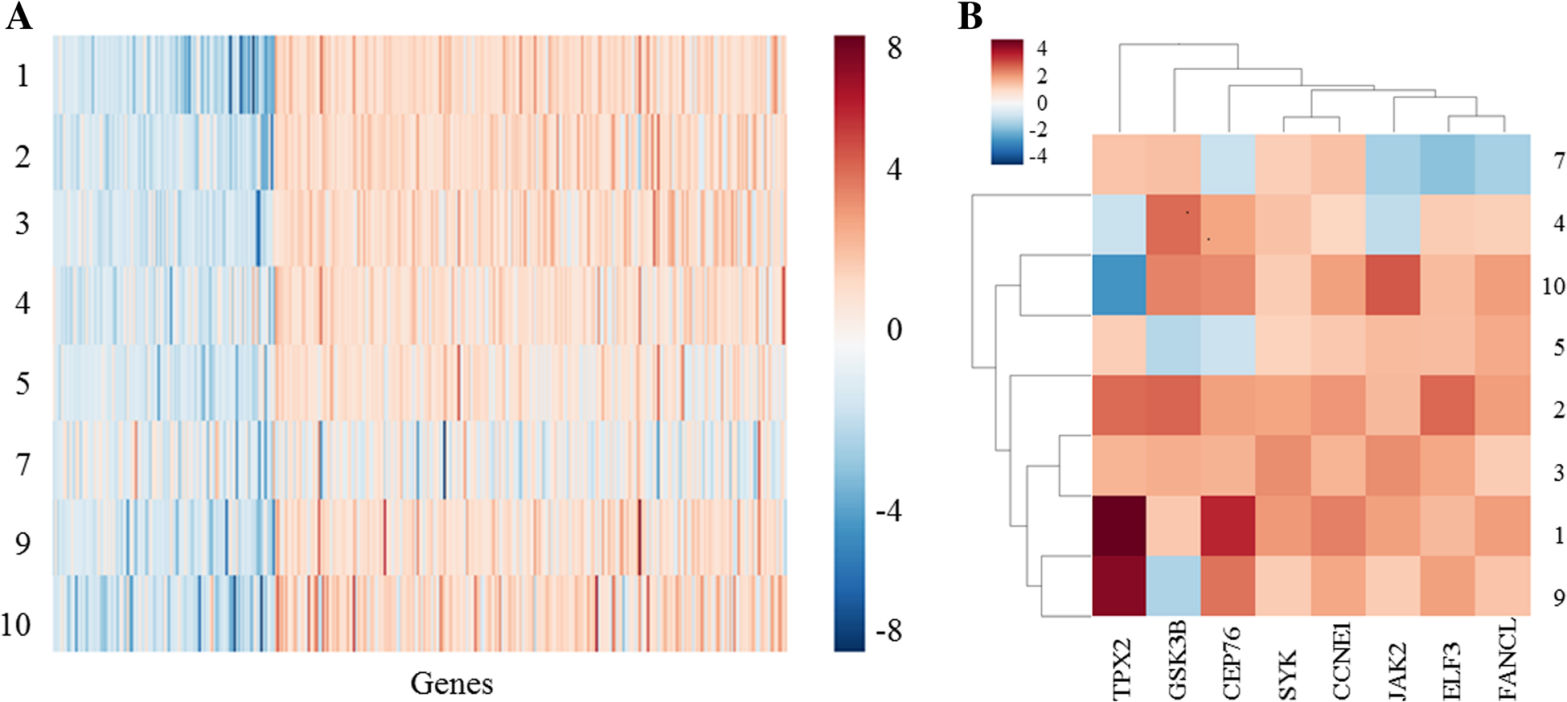
Differential mRNA gene expression analysis between matched primary and metastatic tumors in “Pilot-TNBC” patients (*n* = 8). **a** Heatmap illustrating mRNA expression fold changes (metastatic versus primary, > 1.5) of 455 differentially expressed genes. Three hundred seventeen genes were upregulated and 138 genes were downregulated in metastatic disease versus primary disease. **b** Heatmap highlighting the eight putative cancer driver genes that were upregulated in metastatic TNBCs: *CCNE1, TPX2, GSK3B, CEP76, SYK, JAK2, ELF3* and *FANCL*

To identify genes driving cancer recurrence, gene expression patterns were studied by comparing the paired TNBCs in “Pilot-TNBC.” We found 455 differentially expressed genes (DEG) between metastatic/primary TNBCs (> 1.5 fold changes), with 317 genes upregulated and 138 genes down-regulated in the metastatic TNBCs compared to their matched primaries (Fig. 2a). From the 455 DEGs, the expression of 8 cancer-related genes (*CCNE1, TPX2, GSK3B, CEP76, SYK, JAK2, ELF3* and *FANCL*) was significantly upregulated in recurrent/metastatic TNBCs compared with matched primaries (Fig. 2b). These genes represent potential cancer driver genes, selected based on putative cancer driver gene lists from literature [27, 30–34] and/or inclusion in the FoundationOne™ targeted sequencing panel. There was no clear association between expression pattern of these genes and Lehmann/Pietenpol subtypes of TNBC (data not shown).

### *CCNE1* and *TPX2* mRNA expression in primary breast cancer

**Fig. 3:**
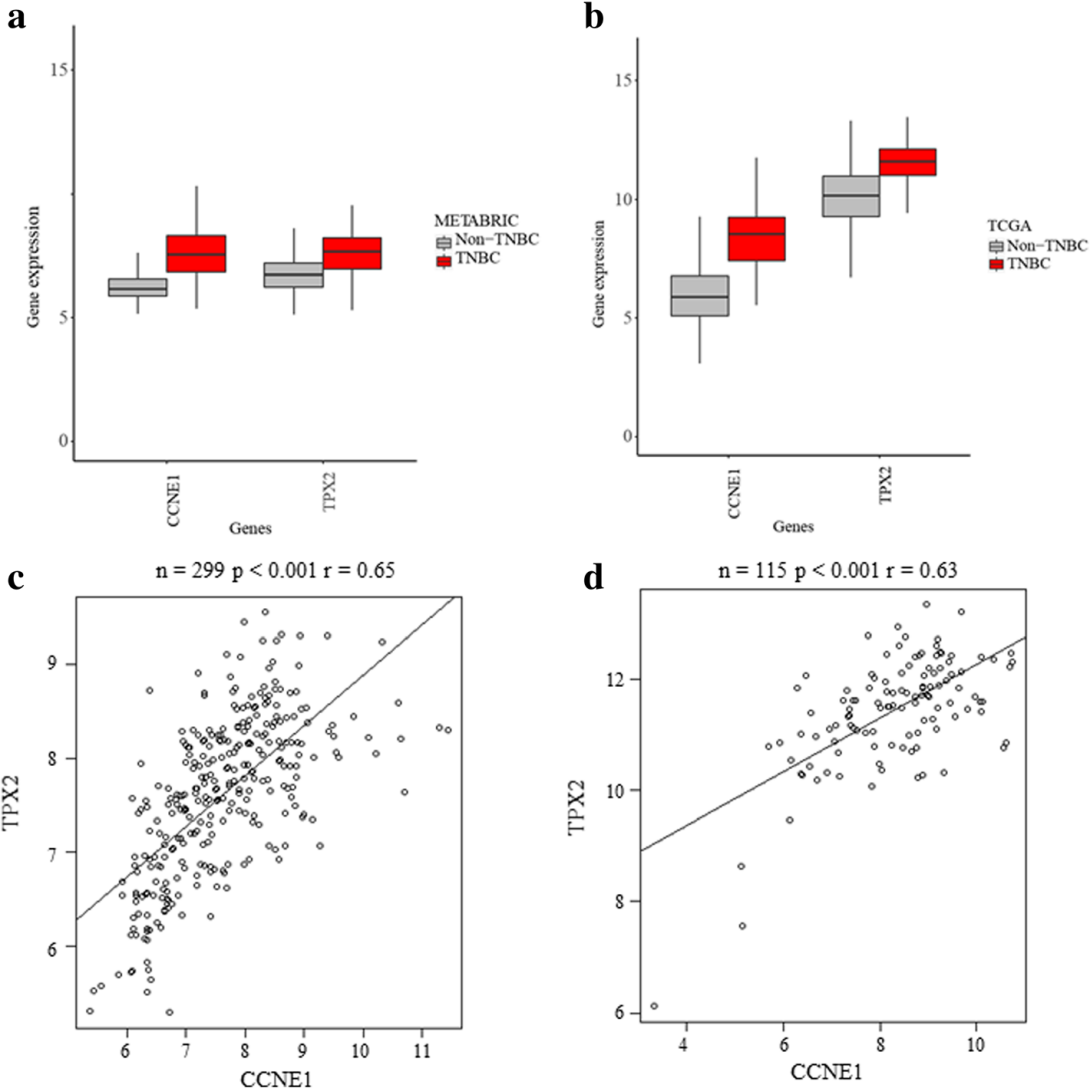
TNBCs exhibited higher mRNA expression of *CCNE1* and *TPX2* compared with non-TNBCs (Wilcoxon signed rank test) in (**a**) METABRIC: TNBC (*n* = 299) and non-TNBC (*n* = 1605) (*p* < 0.0001 for both *CCNE1* and *TPX2*) and in (**b**) TCGA: TNBC (*n* = 115) and non-TNBC (*n* = 976) (*p* < 0.0001 for both *CCNE1* and *TPX2*), y-axis is the log2 of TPM values by RSEM. In TNBCs, *CCNE1* is significantly co-overexpressed with *TPX2* in (**c**) METABRIC: TNBC (*n* = 299, *p* < 0.001, *r* = 0.65) and in (**d**) TCGA: TNBC (*n* = 115, *p* < 0.001, *r* = 0.63)

We next analyzed whether genes upregulated in TNBC metastases had systematic expression patterns across breast cancer datasets. To further understand genomic and transcriptomic expression of *CCNE1* and *TPX2* in primary TNBCs, public databases including METABRIC and TCGA were used. TNBCs exhibited higher mRNA expression of both genes compared with non-TNBCs. In METABRIC, the percentage of tumors with *CCNE1* mRNA overexpression (above median) is significantly increased in TNBCs (42.1%, 126/299) compared with non-TNBCs (3.2%, 51/1605) (*p* < 0.0001). In TCGA, similar results were found in TNBCs (48.7%, 56/115) compared with non-TNBCs (7.2%, 70/976) (*p* < 0.0001). In METABRIC, TNBCs also exhibited higher *TPX2* mRNA overexpression (22.4%, 67/299) compared with non-TNBC (2.4%, 38/1605) (*p* < 0.001). In TCGA, similar results were found for *TPX2* mRNA overexpression in TNBCs (40.9%, 47/115) compared with non-TNBCs (7.6%, 74/976) (*p* < 0.0001) (Fig. 3a and b). In addition, *CCNE1* and *TPX2* are co-overexpressed in TNBC (METABRIC, *n* = 299, *p* ≤ 0.001; TCGA, *n* = 115, *p* ≤ 0.001) (Fig. 3c and d). Furthermore, *CCNE1* is significantly co-overexpressed with *TPX2* in “Pilot-TNBC” and “Discovery-TNBC” cohorts (*p* < 0.001) (Additional file 2: Figure S2). However, *CCNE1* or *TPX* mRNA overexpression in TNBCs did not confer a difference in OS (data not shown).

In METABRIC, TNBCs also exhibited significantly higher mRNA expression of *FANCL* (*p* < 0.0001), *SYK* (*p* < 0.0001), *ELF3* (*p* < 0.01), *JAK2* (*p* < 0.0001), and *GSK3B* (*p* < 0.0001) compared with non-TNBCs (Additional file 3: Figure S1a). There was no significant change in *CEP76*. In TCGA, TNBCs exhibited significantly higher mRNA expression of *FANCL* (*p* < 0.0001), *SYK* (*p* < 0.0001), and *CEP76* (*p* < 0.0001) compared with non-TNBCs (Additional file 3: Figure S1b). No significant changes in *ELF*, *JAK2* and *GSK3B* were observed. Further, compared with normal tissue, median mRNA expression of these eight genes was significantly elevated in breast cancer tissue **(**Additional file 3: Figure S1c).

### *CCNE1* amplification is associated with poor overall survival in the “Discovery-TNBC” cohort

**Fig. 4:**
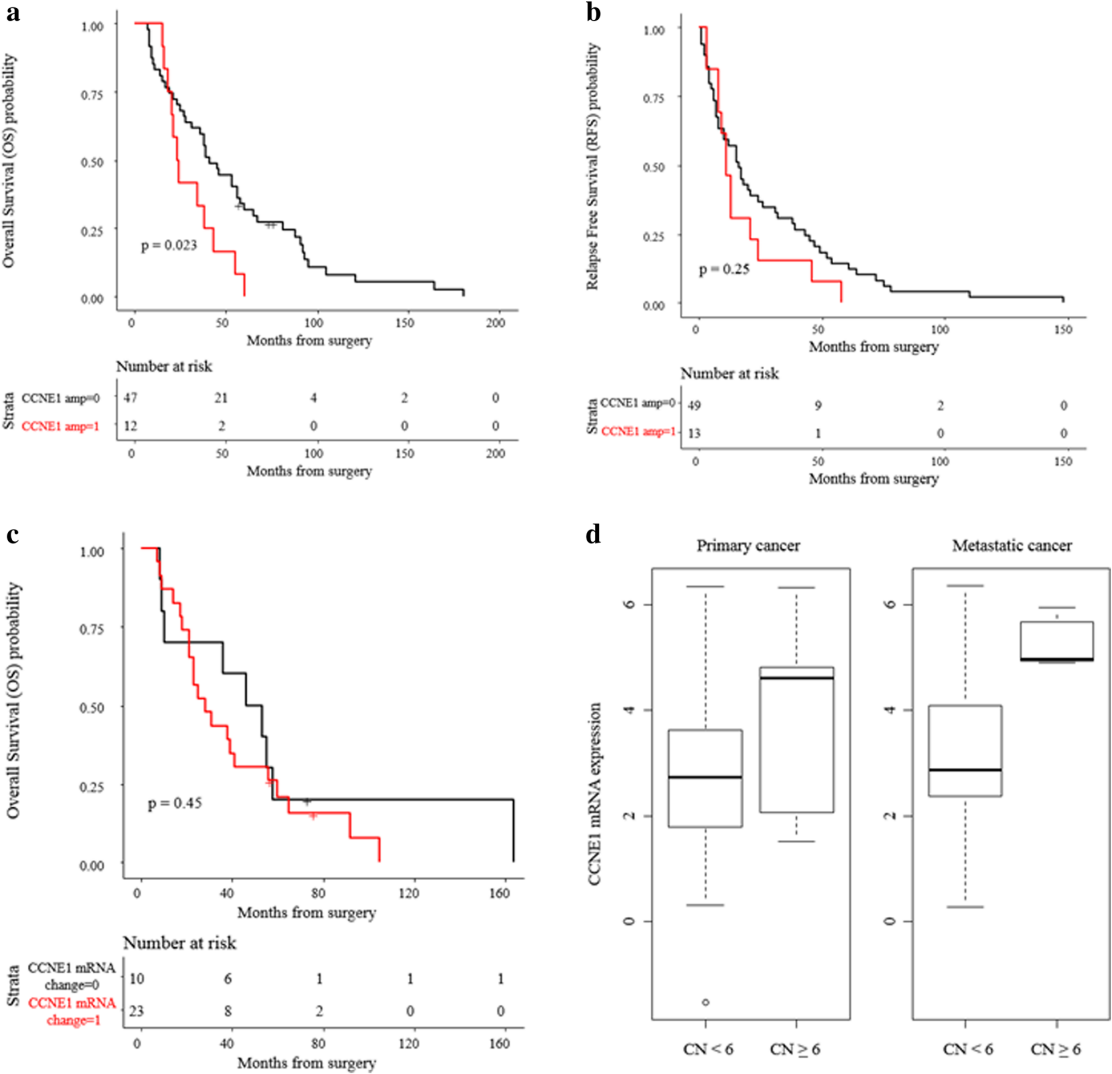
*CCNE1* amplification is associated with poor overall survival (*n* = 62, comprised of 55 patients from the “Discovery-TNBC” cohort and 7 patients from clinical report). (**a**) TNBCs with *CCNE1* amplification (CN > 6) correlate with poor OS (*p* = 0.023); (**b**) No significant correlation was observed between patients’ RFS and *CCNE1* amplification (*p* = 0.25), “CCNE1 amp = 0” represents tumors without CCNE1 amplification (CN < 6), “CCNE1 amp = 1” represents tumors with CCNE1 amplification (CN ≥ 6); (**c**) No significant correlation was observed between patients’ OS and *CCNE1* mRNA expression level (*p* = 0.45), “CCNE1 mRNA change = 0” represents tumors without CCNE1 mRNA overexpression (below median), “CCNE1 mRNA change = 1” represents tumors with CCNE1 mRNA overexpression (above median); (**d**) Analysis of the association of *CCNE1* gene expression and *CCNE1* copy number (CN) alteration in primary and metastatic TNBCs showed that mean and variance of *CCNE1* mRNA expression was comparable between primary and metastatic tumors. Mean *CCNE1* gene expression in metastatic tumors was increased compared to primary tumors (expression = 5.29 vs. 3.87, SD = 0.49 vs. 2.02)

In the “Pilot-TNBC” cohort, *CCNE1* amplification was not observed. Further analysis of an independent cohort of “Discovery-TNBC” revealed *CCNE1* amplification (CN ≥ 6) in 6 of 55 (10.9%) patients (primary and metastatic tumors). The clinical characteristics and outcomes of these patients with *CCNE1*-amplified TNBC are summarized in Table 2. Patients with *CCNE1*-amplified tumors presented with advanced stage disease and had poor pathological responses to neoadjuvant chemotherapy. None of these patients had *BRCA1/2* mutations. There is a statistically significant association between *CCNE1* amplification and poor OS (*p* = 0.023) (Fig. 4a), but not RFS (*p* = 0.25) (Fig. 4b).

**Table 2:** Clinical characteristics and outcomes of patients with *CCNE1*-amplified tumors in the “Discovery-TNBC” cohort

Patient ID	Age	Stage	(Neo)Adjuvant chemotherapy	Response to neoadjuvant chemotherapy	Adjuvant radiation	RFS (months)	OS (months)
1	50	I	Declined	N/A	No	58	N/A
2	50	III	TAC	ypT2N0Mx	No	11	23
3	46	III	AC Carbo/nab-paclitaxel	ypT3N1Mx	Yes	24	55
4	36	III	AC-T	ypT0N1Mx	Yes	13	18
5	35	III	Carbo/taxol	ypT3N2aMx	Yes	3	21
6	46	III	Carbo/taxol	ypT3N3aMx	Yes	13	43
7	50	II	Docetaxel, cisplatin	ypT1N1Mx	Yes	8	15
8	46	III	Carbo/taxol, AC	ypT2N2aMx	Yes	11	20
9	36	II	Carbo/nab-paclitaxel	ypT1cN0Mx	Yes	8	24
10	55	III	AC-T (adjuvant)	pT4N2aMx	No	46	60
11	46	II	Carbo/taxol (adjuvant)	pT2N1Mx	No	21	34
12	53	III	AC-T	ypT1N1aMx	No	9	16
13	48	II	TC	ypT1cN1Mx	Yes	3	38

*AC* adriamycin/cyclophosphamide, *AC-T* adriamycin/cyclophosphamide followed by paclitaxel, *Carbo/taxol* carboplatin/paclitaxel, *TAC* docetaxel/adriamycin/cyclophosphamide, *TC* docetaxel /cyclophosphamide, *RFS* relapse-free survival (surgery to first relapse), *OS* overall survival (surgery to death)

Of the “Discovery-TNBC” cohort (*n* = 55), whole-transcriptome sequencing (RNAseq) was successful in 35 paired primary-metastatic specimens. Of those, 25 specimens exhibited an increase in *CCNE1* mRNA expression in metastatic samples compared to their primary counterparts. No association between *CCNE1* mRNA over-expression and OS was observed in the “Discovery-TNBC” cohort (*p* = 0.45) (Fig. 4c).

We further examined the association of *CCNE1* gene expression and *CCNE1* gene amplification in both primary and metastatic tumors (Fig. 4d). In tumors with no *CCNE1* amplification (CN < 6), the mean and variance of *CCNE1* mRNA expression were comparable between primary and metastatic tumors. However, the mean *CCNE1* expression in metastatic tumors was increased compared to primary tumors (expression = 5.29 vs. 3.87, SD = 0.49 vs. 2.02).

*CCNE1* amplification was more frequently detected in TNBCs (9%, 27/299 in METABRIC and 10%, 11/111 in TCGA) compared to non-TNBCs (1.7%, 27/1605 in METABRIC, *p* < 0.0001 and 3.5%, 33/962 in TCGA, *p* = 0.0055), but these distinctions did not translate into significant OS differences, likely due to limited sample sizes. In TCGA, *TPX2* amplification was more frequent in TNBCs (8.1%, 9/111) compared to non-TNBCs (1.5%, 14/962) (*p* = 0.0004), but not in METABRIC. Interestingly, reflective of our “Discovery-TNBC” cohort data, *CCNE1* amplification in TNBC is mutually exclusive with *BRCA1/2* mutations in both METABRIC and TCGA breast cancer databases.

## Discussion

Tumor genomic evolution is frequently observed due to selection pressure imposed by chemotherapy. We hypothesize that studying paired primary-metastatic TNBCs at the genomic and transcriptomic levels may lead to a better understanding of the underlying mechanisms behind chemotherapy resistance. Herein, we report mRNA expression profiling and targeted DNA sequencing data from paired primary-metastatic TNBCs in a “Pilot-TNBC” cohort and an independent, expanded “Discovery-TNBC” cohort. Despite largely concordant gene expression levels, 8 putative cancer driver genes were significantly upregulated in metastatic samples compared to their matched primary specimens. Furthermore, *CCNE1* amplification was associated with poor overall survival in the “Discovery-TNBC” cohort, highlighting *CCNE1* amplification as a possible mechanism of chemotherapy resistance.

TNBCs are extremely heterogeneous. In an effort to better understand and categorize TNBCs, several molecular classifiers were developed (i.e. Lehmann/Pietenpol [3] and Burstein [4]). Although these classifiers have improved our understanding of TNBC tumor biology and helped distill scientific analyses into outputs that are more easily interpretable, none have led to changes in clinical practice. Herein, we identified so-called “molecular phenotype shifts” between select primary and metastatic samples from our “Pilot-TNBC” cohort, but a universal shift in TNBC pheno-subtypes was not observed. The clinical application of these findings requires further investigation.

Previous studies have reported variable findings upon comparison of primary and metastatic tumors. Some revealed concordant expression patterns between primary breast cancers and their matched synchronous lymph nodes [16, 35–37]. Vecchi, et al. found that expression of a specific gene set could differentiate between primary tumors and synchronous metastases [38]. However, Weigelt, et al. studied seven cases of primary breast cancer and asynchronous distant metastases and showed that a 70-gene prognostic signature was generally maintained in the switch from primary to metastasis across most of the pairs [39]. Several studies used targeted NGS to address genomic concordance between primary and metastases. Meric-Bernstam and colleagues reported that 86.6% of the somatic mutations and 62.3% of the copy number variations were concordant between primary tumors and recurrences [7]. These studies suggest that it may be necessary to study circulating tumor DNA (ctDNA), which is thought to be more representative of the mutational spectrum of the entirety of a patient’s metastatic disease rather than a biopsy of a single metastatic lesion [40]. Furthermore, noncoding RNAs and epigenetic modifications could also play an important role in the metastatic process [16, 41, 42].

Cyclin E and its associated CDK2 are essential for cellular progression through the G1 phase of the cell cycle and initiation of DNA replication. *CCNE1* amplification induces chromosome instability through persistent DNA replication and centrosome duplication [43–45]. CCNE1, along with its catalytic subunit CDK2, plays a critical role in cell cycle regulation to assure precise control of DNA replication, chromosome segregation and the G1 to S-phase transition [11, 12]. *CCNE1* amplification is mutually exclusive with *BRCA1/2* mutations and correlates with cyclin E1 protein expression in ovarian cancer [46–48]. *CCNE1* amplification occurs in approximately 20% of high-grade serous ovarian carcinomas (HGSCs) [13, 14] and is associated with primary treatment resistance and poor outcome in these tumors [49]. Cyclin E1-specific pharmacological inhibitors are not yet available, although indirect CCNE1 targeting trials are ongoing. However, small molecule inhibitors do exist for *CCNE1’s* counterpart in the cell cycle, CDK2. The pan CDK inhibitor, dinaciclib (SCH-727965), inhibits CDKs 2/5/1/9 and is being tested in clinical trials for hematological and solid malignancies (NCT00798213 and NCT00937937) [50, 51]. In addition, a WEE1 inhibitor AZD1755 is being tested in solid tumors with *CCNE1* amplification (NCT03253679). In a study of HGSC, expression of *TPX2*, a centromeric protein required for mitotic spindle function, was highly associated with *CCNE1* amplification [47]. In our study, *CCNE1* and *TPX2* were co-overexpressed in metastatic TNBCs compared to paired primaries. This finding is similar to previous results in ovarian cancer, where co-overexpression of *CCNE1* and *TPX2* were found to be related to clonal resistance to chemotherapy [47]. Mutual exclusivity of *BRCA1/2* mutation/deletion and *CCNE1* amplification has been shown previously in ovarian cancers [47]. Despite limited sample size, our finding of mutual exclusivity of *CCNE1* and *BRCA1/2* genes is worth further investigation and may help clinicians select patients for personalized treatment.

High cyclin E1 expression has been considered in other studies as a biomarker of poor clinical outcome in breast cancer [15, 52]. In a study of patients with HER2+ disease, *CCNE1* amplification/overexpression resulted in worse survival and CDK2 inhibition could overcome trastuzumab resistance in xenograft models [15]. Hunt, et al. demonstrated that overexpression of cytoplasmic low molecular weight cyclin E (LMW-E) is associated with poor survival in patients with breast cancer [53]. Because it lacks a nuclear localization domain, LMW-E accumulates in the cytoplasm, where it binds to CDK2 and retains kinase activity [54]. Our study provides further evidence of the association of *CCNE1* amplification and poor OS in patients with recurrent TNBCs. *CCNE1* mRNA was significantly upregulated in metastatic compared to paired primary TNBCs. Although no survival differences were observed, this may be reflective of the limited sample size.

This study features a combination of well-annotated clinicopathologic and genomic data, which allowed us to directly identify clinically relevant genomic information. However, our study is limited by sample size, lack of unified treatment variables, and retrospective nature. We did not study noncoding RNAs nor epigenetic changes within the tumors. Finally, although patient-matched, singular biopsies do not represent a complete picture of metastatic disease. Overall, our study is hypothesis-generating and these results will benefit from verification with a larger cohort of patients and more comprehensive technologies and tumor biology assessments such as ctDNA analyses.

## Conclusions

In this study, comparison of paired primary and metastatic TNBCs demonstrates heterogeneity of molecular mechanisms of gene expression underlying TNBC recurrence. Amplification of *CCNE1* is associated with poor OS in patients with metastatic TNBC. *CCNE1* amplification may serve as a target for therapeutic intervention in chemotherapy-resistant TNBCs.

## Additional files

Additional file 1: **Table S3.** Genomic alteration data for the “Pilot-TNBC” cohort. (XLSX 39 kb)

Additional file 2: **Figure S2.** *CCNE1* is significantly co-overexpressed with *TPX2* in “Pilot-TNBC” and “Discovery-TNBC” cohorts (*p* < 0.001). (TIF 165 kb)

Additional file 3: **Figure S1.** TNBCs exhibited higher mRNA expression compared with non-TNBCs for the eight putative cancer driver genes identified in the differential expression analysis in (a) METABRIC (TNBC, *n* = 299 and non-TNBC, *n* = 1605) and (b) TCGA (TNBC, *n* = 115 and non-TNBC, *n* = 976). Analysis-values were calculated using the Wilcoxon test (*p < 0.01, **p < 0.0001); (c) TNBCs also exhibited higher mRNA expression compared to non-TNBCs and normal breast tissue for the eight putative cancer driver genes in TCGA (TNBC, n = 115; non-TNBC, *n* = 976; and normal breast, *n* = 112) as per the ANOVA test (*p < 0.01, **p < 0.0001), y-axis is the log2 of TPM values by RSEM. (TIF 120 kb)

Additional file 4: **Table S1.** Normalized mRNA expression data for the “Pilot-TNBC” cohort. (XLSX 13 kb)

Additional file 5: **Table S2.** Normalized mRNA expression data for the “Discovery-TNBC” cohort. (XLSX 20 kb)

## Abbreviations

AC: Adriamycin/cyclophosphamide
AC-T: Adriamycin/cyclophosphamide followed by paclitaxel
BC: Breast cancer
BL1: Basal-like 1
BL2: Basal-like 2
Carbo/paclitaxel: Carboplatin/paclitaxel
*CCNE1*: Cyclin E1
cfDNA: Cell-free DNA
DEG: Differentially expressed genes
ER: Estrogen receptor
Exome-Seq: Exome-sequencing
FDR: False discovery rate
HER2+: Human epidermal growth factor receptor 2 positive
HGSCs: High-grade serous ovarian carcinomas
HQ: Highest quartile
HR+: Hormone-receptor positive
IDC: Invasive ductal carcinoma
ILC: Invasive lobular carcinoma
K-M: Kaplan-Meier
LAR: Luminal androgen receptor
LN: Lymph node
LQ: Lowest quartile
M: Mesenchymal
met: Metastatic tumor
METABRIC: Molecular Taxonomy of Breast Cancer International Consortium
mRNA: Messenger RNA
OS: Overall survival
PR: Progesterone receptor
RFS: Relapse-free survival
RMA: Robust multi-array average
TAC: Docetaxel/adriamycin/cyclophosphamide
TC: Docetaxel (Taxotere) /cyclophosphamide
TCGA: The Cancer Genome Atlas
TNBC: Triple negative breast cancer
VUS: Variants of unknown significance
