# Supplementary figures and images for: CCNE1 amplification is associated with poor prognosis in patients with triple negative breast cancer
Source: BMC Cancer. 2019 Jan 21;19:96. doi: 10.1186/s12885-019-5290-4 (PMC6341717; doi:10.1186/s12885-019-5290-4)

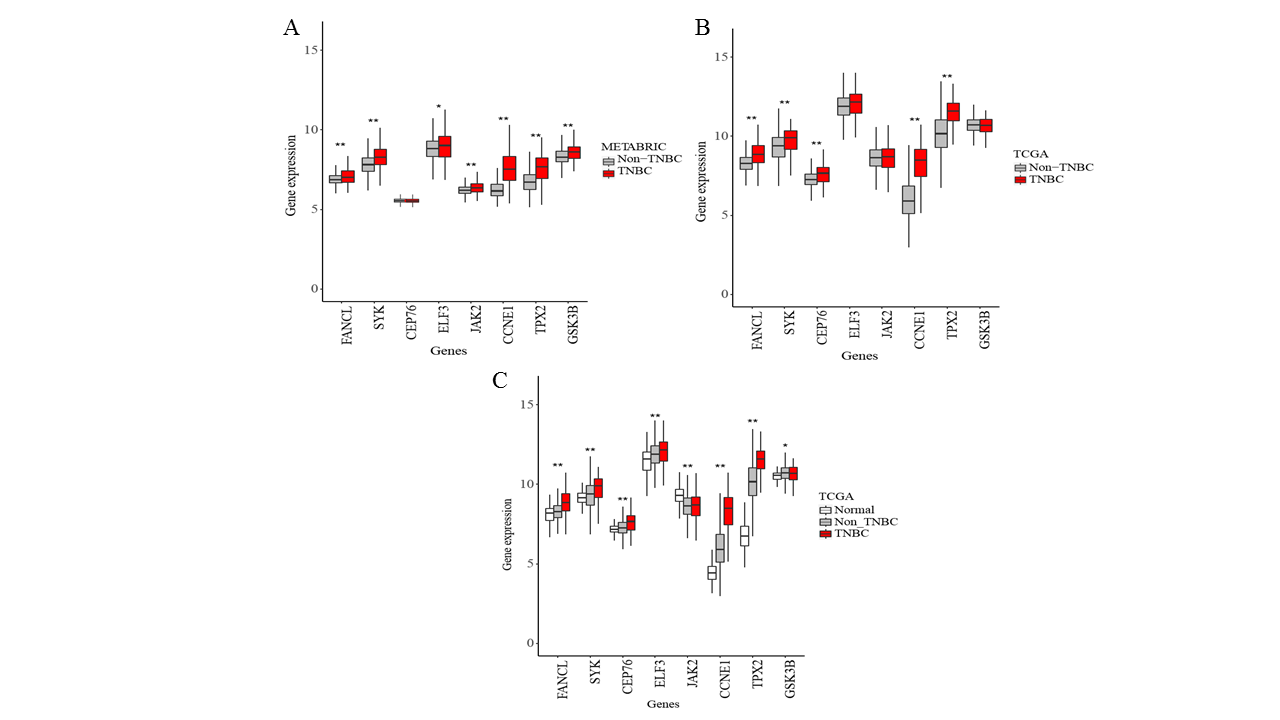

Supplement: Supplementary file 2 — Figure S2. CCNE1 is significantly co-overexpressed with TPX2 in “Pilot-TNBC” and “Discovery-TNBC” cohorts (p < 0.001). (TIF 165 kb) [file 12885_2019_5290_MOESM2_ESM.tif]

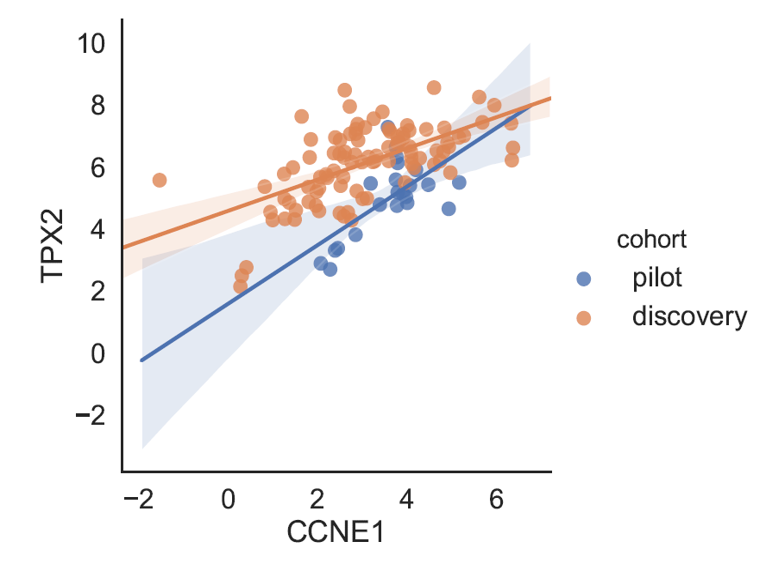

Supplement: Supplementary file 3 — Figure S1. TNBCs exhibited higher mRNA expression compared with non-TNBCs for the eight putative cancer driver genes identified in the differential expression analysis in (a) METABRIC (TNBC, n = 299 and non-TNBC, n = 1605) and (b) TCGA (TNBC, n = 115 and non-TNBC, n = 976). Analysis-values were calculated using the Wilcoxon test (*p < 0.01, **p < 0.0001); (c) TNBCs also exhibited higher mRNA expression compared to non-TNBCs and normal breast tissue for the eight putative cancer driver genes in TCGA (TNBC, n = 115; non-TNBC, n = 976; and normal breast, n = 112) as per the ANOVA test (*p < 0.01, **p < 0.0001), y-axis is the log2 of TPM values by RSEM. (TIF 120 kb) [file 12885_2019_5290_MOESM3_ESM.tif]
